# Negative auto-regulation increases the input dynamic-range of the arabinose system of *Escherichia coli*

**DOI:** 10.1186/1752-0509-5-111

**Published:** 2011-07-12

**Authors:** Daniel Madar, Erez Dekel, Anat Bren, Uri Alon

**Affiliations:** 1 Department of Molecular Cell Biology, The Weizmann Institute of Science, Rehovot, 76100, Israel

## Abstract

**Background:**

Gene regulation networks are made of recurring regulatory patterns, called network motifs. One of the most common network motifs is negative auto-regulation, in which a transcription factor represses its own production. Negative auto-regulation has several potential functions: it can shorten the response time (time to reach halfway to steady-state), stabilize expression against noise, and linearize the gene's input-output response curve. This latter function of negative auto-regulation, which increases the range of input signals over which downstream genes respond, has been studied by theory and synthetic gene circuits. Here we ask whether negative auto-regulation preserves this function also in the context of a natural system, where it is embedded within many additional interactions. To address this, we studied the negative auto-regulation motif in the arabinose utilization system of *Escherichia coli*, in which negative auto-regulation is part of a complex regulatory network.

**Results:**

We find that when negative auto-regulation is disrupted by placing the regulator *araC *under constitutive expression, the input dynamic range of the arabinose system is reduced by 10-fold. The apparent Hill coefficient of the induction curve changes from about *n *= 1 with negative auto-regulation, to about *n *= 2 when it is disrupted. We present a mathematical model that describes how negative auto-regulation can increase input dynamic-range, by coupling the transcription factor protein level to the input signal.

**Conclusions:**

Here we demonstrate that the negative auto-regulation motif in the native arabinose system of *Escherichia coli *increases the range of arabinose signals over which the system can respond. In this way, negative auto-regulation may help to increase the input dynamic-range while maintaining the specificity of cooperative regulatory systems. This function may contribute to explaining the common occurrence of negative auto-regulation in biological systems.

## Background

**Figure 1:**
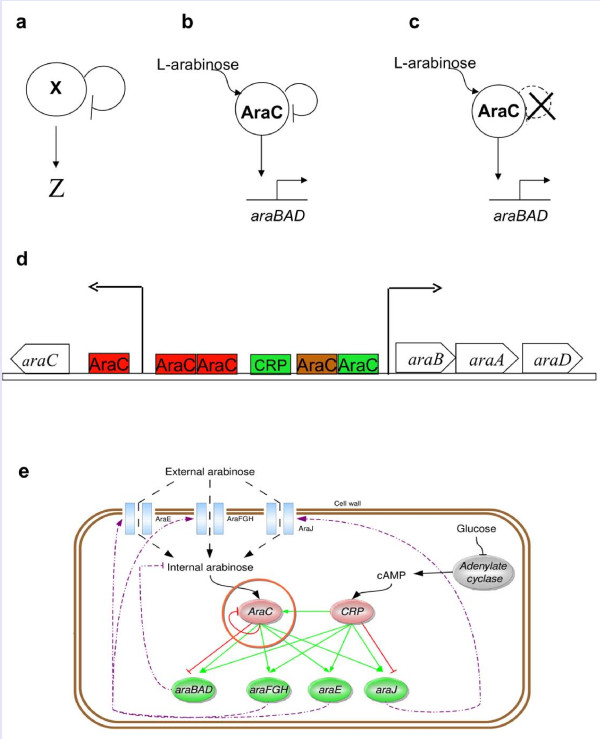
**An overview of the regulatory network of the arabinose utilization system in *E. coli***. **(a)**. NAR motif with transcription factor X that regulates its own production, and also regulates the production of gene Z (Z often represents several different downstream promoters). **(b) **The *araBAD *genes are regulated by AraC which is negatively auto-regulated. **(c) **NAR in this study was disrupted by decoupling *araC *from its native regulation. **(d) **The *araC*\*araBAD *divergent promoter structure. Colorless boxes are genes and colored boxes are the transcription factors' binding sites: red- repressor; green- activator; brown- dual regulator (based on Ecocyc [50,51], see text for details). **(e) **The arabinose system is a complex regulatory network in which NAR is only one of many interaction arrows, in which transporters and enzymes modify the intracellular arabinose levels, which in turn acts to repress and activate the pumps and enzymes genes (see text for details).

Transcription regulation networks are largely made up of recurring regulatory patterns called network motifs [1-4]. These network motifs have been demonstrated to carry out specific information-processing functions (e.g. [1,3,5]). One of the simplest and most abundant network motifs is negative auto-regulation (NAR). In this motif, a transcription factor (TF) negatively regulates the promoter of its own gene or operon [1,3,6] (Figure 1a). Approximately 40% of the known transcription factors in *Escherichia coli *show negative auto-regulation [7], as do many transcription factors in yeast and higher organisms [3,8-11].

NAR has been suggested experimentally and theoretically to have several functions. The first is increased homeostasis or buffering of the auto-regulated gene product concentration against stochastic noise [12-14]. Because protein levels can vary from cell to cell by tens of percents [15-17], such a noise buffering mechanism is useful in cases where precision in TF levels is needed [18]. Low frequency noise in TF production rates tends to be buffered by NAR because negative feedback reduces TF levels if they are too high, and increases them if they are too low, making TF levels more uniform across cells.

A second feature of NAR is its ability to speed the response time of gene circuits [6,19,20]. Response time is defined as the time it takes to reach half of the total change in a dynamic process. Theoretical comparison between NAR and a simply regulated promoter with no NAR, with parameters in which both reach the same steady-state level expression, shows that the response time is shorter when the TF is negatively auto-regulated. This speed up is achieved by the use of a strong promoter allowing a rapid initial rise in TF levels, up to its auto-repression threshold, followed by reduction in production rate due to NAR [19]. This speedup feature was observed in a synthetic NAR circuit [19] as well as in the native SOS DNA repair system of *E. coli *[20]. Speedup offered by NAR may be advantageous in dynamic environments where rapid responses improve fitness.

Recently, it was shown by Nevozhay *et al*. that NAR can also linearize dose responses [21]. In this study the response of synthetic, TetR-based transcriptional circuits with and without NAR was studied in *S. cerevisiae *as a function of inducer (anhydrotetracycline, aTc) levels. NAR was found to transform a sigmoid induction curve into a more linear curve (see also [18]). This feature was also suggested in theoretical studies [6,22-25]. This role of NAR can be interpreted as an *increase in the input dynamic range *- the range of input signals over which the system can respond.

Such theoretical and synthetic-circuit studies are a powerful approach because one can study the function of circuits such as NAR without of interfering effects. In natural systems, however, this motif is embedded inside a large regulatory network with many other interactions. These additional interactions might in principle modify its function. Therefore, to fully test the function of a motif requires, in addition to theory and synthetic circuits, experiments on the motif in its natural context, wired into the full interaction networks of the cell.

Here, we study the function of the NAR motif in a natural system. We chose one of the best-studied gene regulation systems, the arabinose utilization system of *E. coli*. This system has been characterized over the past decades by Schleif and colleagues ([26-29] for reviews). The arabinose-responsive TF, called AraC, is negatively autoregulated (Figure 1b). We asked whether NAR increases the input dynamic range in this system.

The arabinose system is composed of 9 genes arranged in 5 operons: *araC*- the system-specific TF; *araE, araFGH, araJ*- the arabinose transporters [30-32]; and *araBAD*- arabinose catabolic enzymes. Two operons, *araC *and *araBAD*, are divergent and share the same regulatory region (Figure 1d). The system is regulated by cAMP Receptor Protein (CRP) and AraC (Figure 1e), which are activated by cAMP and L-Arabinose respectively [26-29]. AraC represses its own promoter, creating a NAR motif. It both activates and represses the arabinose utilization operon *araBAD *by means of a DNA looping mechanism [33,34]. AraC undergoes a conformational change when it binds L-Arabinose, leading to expression of the *ara *genes. The system includes several interactions and feedback loops, in which metabolic enzymes and transporters downstream of *araC *affect the level of intracellular arabinose, the inducer that activates AraC (Figure 1e). In a study of the input functions of *E. coli *sugar systems, it was recently found that promoters from the arabinose system respond to their inducer with a wider input dynamic range compared to other sugar systems (eg. the maltose system) in which the TF is not negatively auto-regulated [35].

To test the role of NAR, we compared the wild type *ara *system (Figure 1b) to a variant in which NAR is disrupted by placing the regulator AraC under a constitutive promoter (Figure 1c). We find, using high-temporal resolution measurement of promoter activity, that disrupting NAR in the arabinose system increases the steepness of the sigmoidal response curve. It reduces the input dynamic range by about an order of magnitude. Thus, NAR increases input dynamic range in the context of the natural *ara *system. We also analyze this mathematically, suggesting that the increase in input dynamic range is due to the increase of AraC protein level with increasing arabinose due to the NAR.

## Results

### The native input function of the *araBAD *promoter has a broad input dynamic range

**Figure 2:**
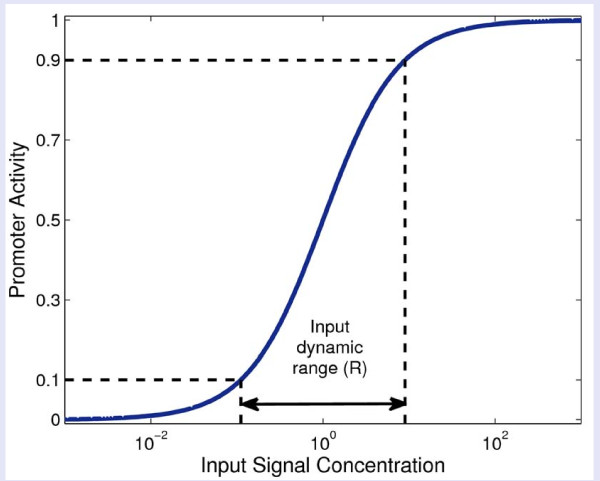
**Gene input function and its input dynamic range**. The input function is defined as the normalized promoter activity at different signal concentrations. The black horizontal dashed lines mark the 10% and 90% promoter activity. The input dynamic range is the ratio *R *of input concentrations required for 90% and 10% of maximal output.

The input dynamic range is defined as the range of inputs over which the output changes significantly. Operationally, following Goldbeter and Koshland [36-38], we define the input dynamic range as the ratio *R *of input levels at which the system shows 90% and 10% of its maximal output (Figure 2). For a Hill curve with coefficient *n*, the input dynamic range is *R *= 81^1/n^. Thus, Michaelis-Menten like curves with *n *= 1 show *R *= 81, steeper sigmoidal curves with *n *= 2 show *R *= 9, and very steep cooperative curves with *n *= 4 show *R *= 3.

In order to determine the input dynamic range of *E. coli *promoters we used a fluorescent reporter automated assay [35,39], with strains from the comprehensive *E. coli *transcription reporter library [40]. Each strain bears a low-copy plasmid with a green fluorescent protein gene under the control of a full length copy of the promoter of interest. In this study we used reporters for the *araBAD *and *araC *promoters in *E. coli *strain MG1655 (see Methods).

Reporter strains were grown on glucose minimal medium containing saturating amount of cAMP (30 mM, to fully activate CRP) and increasing amounts of L-Arabinose [35,41]. Promoter activity (PA) was defined as the rate of GFP production per OD (optical density) unit, PA = dGFP/dt/OD (see Methods). The input functions were derived from the promoter activity averaged over a window that spans 1-2 cell generations in exponential phase (5-7 hours after initial 1:600 inoculation). Over this time window, promoter activity was constant to a good approximation (see Additional File 1 for fluorescence and growth curves, p. 2-3, Figure S1 and S2 respectively).

**Figure 3:**
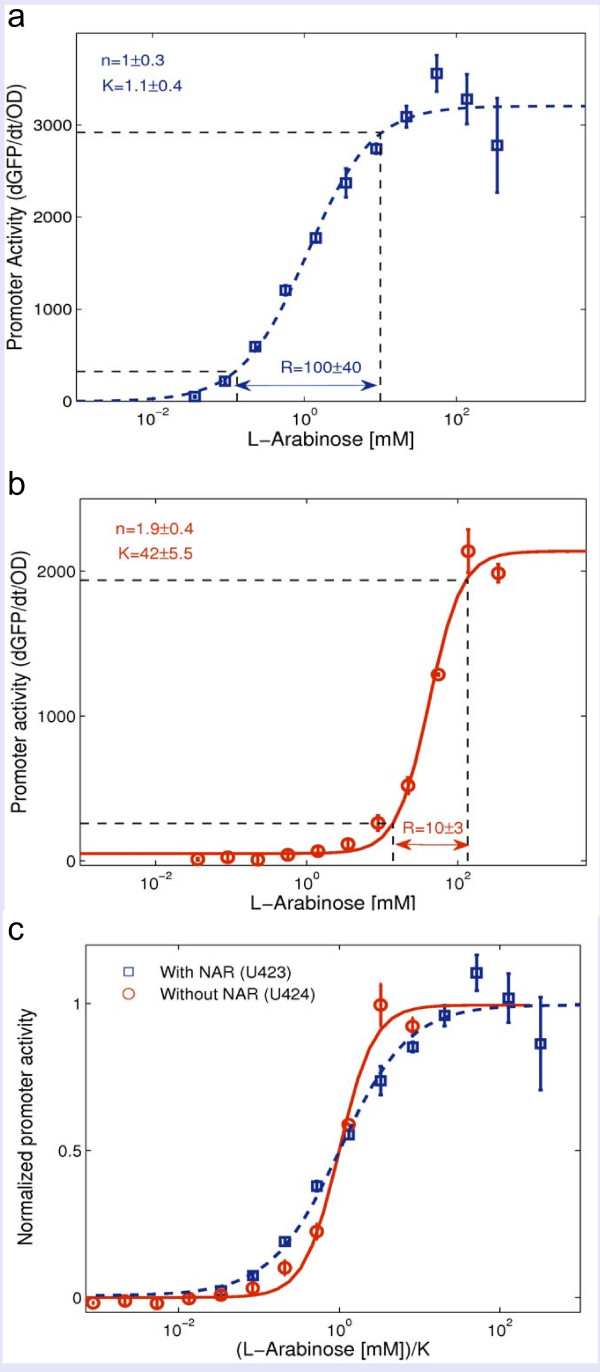
**The input dynamic range of the *araBAD *operon is reduced by disrupting the NAR that controls *araC***. Shown is *araBAD *promoter activity as a function of arabinose concentration. The error bars indicate the s.e. **(a) **Promoter activity (dGFP/dt/OD, arbitrary units) in the parental strain (U424, with NAR). The blue squares are the experimental results. The dashed blue line is a fitted Hill function with Hill coefficient *n *= 1 ± 0.3 (s.e.), *K *= 1.1 ± 0.4 mM (s.e.), and *R *= 100 ± 40 (s.e.), and also the best fit solution of the full model described in Additional File 1. The black horizontal dashed lines mark the 10% and 90% promoter activity. **(b) **Promoter activity (dGFP/dt/OD, arbitrary units) in the mutant strain (U426, without NAR). The red circles are the experimental results. The solid red line is a fitted Hill function with Hill coefficient *n *= 1.9 ± 0.4 (s.e.), *K *= 42 ± 6 mM (s.e.), and *R *= 10 ± 3 (s.e.), and also the best fit solution of the full model described in Additional File 1. The black horizontal dashed lines mark the 10% and 90% promoter activity. **(c) **Normalized promoter activity of the two strains. The x axis is the L-Arabinose concentration divided by *K *per strain. This best demonstrates differences in the input dynamic range (*R*) between the two strains. Blue squares and dashed blue line are of the parental strain (U424, with NAR), while red circles and solid red line are of the mutant strain (U426, without NAR). Symbols are the measured results and the lines are fitted Hill functions.

The promoter activity of the *araBAD *in the parental strain (wild-type *araC *regulation, U424) as a function of arabinose concentration is shown in Figure 3a. At low arabinose levels (below about 10 μM arabinose) the fluorescence of the reporter is indistinguishable from the cells auto-fluorescence background. The input function reaches 10% of its maximal value at arabinose levels of about 0.1 mM, and 90% of its maximal value at about 10 mM. Fitting a Hill curve to the input function results in an apparent Hill coefficient of *n *= 1 ± 0.3 (s.e.), and halfway induction point of *K *= 1.1 ± 0.4 mM (s.e.). The input dynamic range is *R *= 100 ± 40 (s.e.). These results are similar to measurements of the input function of the *araBAD *reporter strain in wild-type MG1655 (U429) [35], and are consistent with the expected value for a curve with Hill coefficient equal to *n *= 1, in which *R *= 81.

### The *araC *gene is induced by arabinose

**Figure 4:**
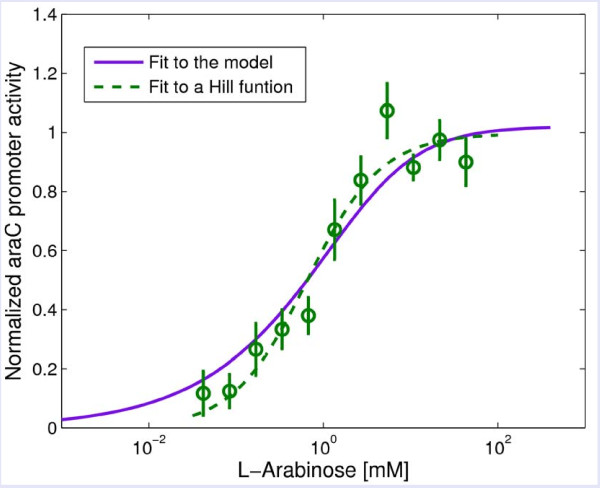
**The promoter activity of *araC *increases with arabinose**. Shown is *araC *promoter activity as a function of arabinose concentration, in the wild-type strain (with NAR, green circles). The dashed green line is a fitted Hill functions with *n *= 1 ± 0.6, *K *= 0.6 ± 0.4M (s.e.). The solid purple line is the best fit of the full model described in Additional File 1.

We also tested the dependence of the *araC *promoter activity on arabinose. Since AraC negatively regulates its own promoter, arabinose is expected to affect *araC *expression. Indeed, using an *araC *reporter strain (U428), we find that arabinose increases the activity of the *araC *promoter in a dose-dependent manner (Figure 4) [35].

### Disruption of negative auto-regulation of *araC *reduces the input dynamic range of *araBAD*

To study the role of the negative auto-regulation of *araC *on the input dynamic range of its downstream genes, we decoupled *araC *expression from its negative auto-regulation (Figure. 1c). For this purpose we deleted the *araC *open reading frame from the chromosome of the wild-type strain MG1655 and re-introduced *araC *on a plasmid (pZE11) which provides constitutive expression (strain U426, see Methods). The plasmid has a *tetR *controlled promoter, repressed by a chromosomal *tetR *gene. Without induction, this plasmid produces levels of AraC that are comparable to the induced wild-type AraC level, as assessed from the maximal promoter activity of the *araBAD *reporter. It should be noted that the parental strain in this study (U424) also contains chromosomal *tetR *as well as an emptly pZE11 vector, in order to preserve genotypic identity between the two strains.

We find that in the absence of NAR, the arabinose-dependent input function of *araBAD *is significantly steeper than the parental input function (Figure 3b,c), with an apparent Hill coefficient of *n *= 1.9 ± 0.4 (s.e.), and halfway induction point of *K *= 42 ± 0.6 mM (s.e.). The measured input dynamic range spans between 14 mM - 135 mM, and thus has *R *= 10 ± 3 (s.e.), in comparison to *R *= 100 ± 40 (s.e.) in the parental strain. Thus, decoupling *araC *from its negative auto-regulation reduces the input dynamic-range of its downstream genes by about an order of magnitude.

### A model of NAR and increased input dynamic range

What is the main effect at play that allows negative auto-regulation to increase input dynamic range? To understand this, we analyzed a mathematical model of the NAR motif. We sought to make the model as simple as possible, in order to be able to understand it intuitively, and at the same time not too simple so as not to lose the essence of the problem. A more comprehensive model, based on mass-action kinetics, which includes a dual transcription factor that acts as both a repressor and an activator, is given in Additional File 1 (p. 5-7).

Consider a transcription factor whose concentration is *X*, that binds its inducer *s *with a dissociation constant *K*_*s*_. The amount of *X *bound to *s*, which is the active form of the transcription factor X*, is described by Michaelis-Menten binding:(1)

The active transcription factor X* binds the promoter of a downstream gene *Z *with Michaelis-Menten-like kinetics, so that the steady-state level of the *Z *gene product is:(2)

Where *K*_*z *_is the dissociation constant of X^* ^from the promoter of *Z*, *β*_*Z *_is the maximal production rate of *Z*, and *α *is its degradation/dilution rate [1].

Without negative auto-regulation, the concentration of *X *is independent of the inducer levels. We denote this constant level *X*_*0*_. Using Eq. (1) in Eq. (2) with *X = X*_*0 *_results in a sigmoidal regulation function with an input dynamic range of *R *= 9.(3)

It is at this point that negative auto-regulation has an important effect: instead of a constitutive level of *X*, negative auto-regulation allows the signal *s *to modify the concentration of *X*, an effect termed direct coupling [42]. With negative auto-regulation of the type found in the *ara *system, the promoter that encodes *X *is repressed by free *X*, (denoted *X*_*f*_) a repression which is relieved when *X *is bound to the signal.

To analyze this, consider the rate of production of *X *that is repressed by *X*_*f *_[19], balanced by degradation/dilution of the protein at rate *α*, so that:(4)

Where *K*_*x *_is the dissociation constant of *X *from its own promoter, and the free *X *(*X*_*f*_) is given by the unbound fraction, *X*_*f *_= X-X*:(5)

Substituting Eq. (5) into Eq. (4) and assuming strong binding of the regulator to its own promoter *K*_*x*_*<<X*_*f*_, one finds that at steady-state the *level of X increases as the square root of the input signal s*:(6)

Where *A*^*2 *^*= K*_*x*_*β*_*x*_*/α*. In other words, the transcription factor (*X*) levels increases with the signal (*s*) levels (see the relationship between AraC and L-Arabinose in Figure 4).

Using this expression for *X *instead of *X*_*0 *_in Eq. (2), results in an input-function that is less steep, because of the square-root dependence of *X *on *s*:(7)

Where . This function has an input dynamic range of *R *= 81, which is 9 fold wider than that of Eq. (3). Thus, NAR increases the input dynamic range.

Note that the assumption *K*_*x*_*<<X*_*f *_is not crucial for the increased input dynamic range, and was used only for the sake of simplicity. In Additional File 1 we present a full mass-action model, without these assumptions, and show that the present considerations apply as well.

**Figure 5:**
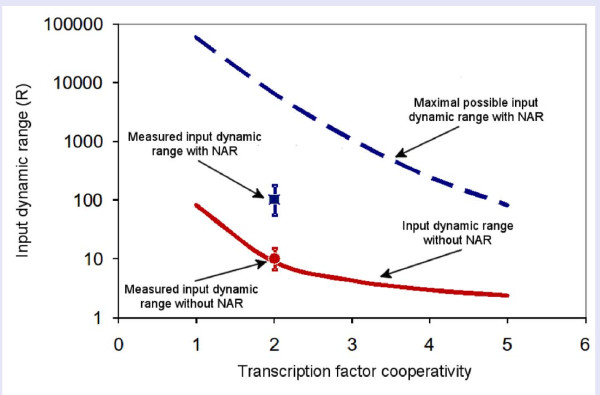
**NAR model suggests increased input dynamic range for regulators with varying degrees of cooperativity**. Cooperativty of the regulator is described by a Hill coefficient for the binding of TF to its downstream promoter. Dashed blue line is the maximal possible input dynamic range that can be reached by a system with a negatively auto-regulated TF, as found by scanning the entire range of model parameters. Solid red line is the input dynamic range that the system displays without negative auto-regulation, given by *R *= 81^(1/n)^. The blue square and red dot are the experimentally measured input dynamic ranges of *araBAD *with and without NAR, respectively.

We further investigated the effect of NAR on input functions with different cooperativity in the binding of the TF to the promoter, as described by Hill equations. In the present system, the *araBAD *input function without NAR has an apparent Hill coefficient of about 2, suggesting that the AraC regulator is cooperative with *n *= 2. In Figure 5 we describe the results of the model with regulators with degrees of apparent cooperativity of the regulator ranging between *n *= 1 and *n *= 5. It is seen that NAR increases the input dynamics range in all cases. For example, at *n *= 2, the input dynamic range without NAR is *R *= 9, but can reach up to *R *~ 1000 with NAR (the values observed above for the *ara *system are about *R *= 10 without NAR and *R *= 100 with NAR).

Furthermore, the model explains how the change in regulator levels caused by NAR can cause a shift in the halfway induction point *K *of downstream genes (relative to no NAR). The direction and size of the shift depends on the mode of regulation. For repressors, *K *generally increases with regulator levels, whereas activators show the converse dependence. Since AraC both activates and represses *araBAD*, the detailed model in Additional File 1 explains the observed increase in *K *shown in Figure 3 (Additional File 1, p.8-9, Figure S3).

To summarize the conclusions of this analysis, negative auto-regulation causes regulator levels to increase with inducer level. This enhances the input dynamic range by extending the range of inputs that can affect the downstream genes.

## Discussion

This study supports a role for negative auto-regulation in increasing the input dynamic range of downstream genes. Previous studies suggested this role theoretically [6,22-24] and demonstrated it using synthetic circuits [18,21]. Here we tested NAR in the context of a natural system, the arabinose system of *E. coli*, in which NAR is embedded within multiple feedback loops and regulatory interactions. Disruption of the NAR in the arabinose system reduced the input dynamic range by an order of magnitude.

What is the intuitive explanation for the enhancement of the input dynamic range provided by negative auto-regulation? Negative auto-regulation in the arabinose system allows the transcription factor concentration to be modulated by its own input signal. As the concentration of input signal increases, the concentration of transcription factor also increases. This extends the response range of downstream promoters, which would otherwise reach maximal activity when the transcription factor becomes saturated with input signal.

A related but distinct feature was studied by M. Savageau [6,42], in which proper coupling of inducer levels and transcription factor levels can increase the output (as opposed to input) dynamic range of genes: the ratio of their maximal to minimal expression level.

Use of NAR to increase input dynamic range might be especially useful for regulators that bind the promoter cooperatively. Such cooperative binding is thought to increase specificity [43]. However, a well-known feature of cooperative binding (high Hill coefficient) is a narrow input dynamic range [43]. NAR is a simple way to provide wide input dynamic range, while maintaining cooperativity at the level of regulator binding. The combination of cooperativity and negative auto-regulation might thus provide a response across several decades of input strength and at the same time remains specific.

## Conclusions

The present study adds to our understanding of the functions of negative auto-regulation network motif, showing that it can increase the input dynamic range of the response, even when embedded in a relatively complicated native gene circuit. Integration of negative auto-regulation within a system with high cooperativity (high specificity and steep activation curve), enables the system to respond to a wide range of input signal (making the activation curve wide) while maintaining the system's specificity to the signal. This function can be experimentally tested in the numerous additional gene systems which bear this network motif across organisms. Because the negative auto-regulation motif is not limited to transcription networks this feature might also apply to other biological systems including protein-level interactions.

## Methods

### Plasmids (see Table 1)

**Table 1:** Plasmids and strains used in this study

Plasmids	Description	Source
pUA66	sc101 *ori*, promoterless version of the GFP reporter plasmid, (kan^R^).	[40]

*P*_*araBAD *_reporter	GFP reporter plasmid for the *araBAD *promoter (*P*_*araBAD *_in pUA66), (kan^R^).	[40]

*P*_*araC *_reporter	GFP reporter plasmid for the *araC *promoter, (*P*_*araC *_in pUA66), (kan^R^).	[40]

pZE11	Control plasmid: colE1 *ori*, *P*_*LtetO-1 *_promoter, (amp^R^) [45].	This study

pZE11-*araC*	*araC *controlled by the *tet *promoter on pZE11, (amp^R^).	This study

**Strains**		

U423	MG1655z1: MG1655 (F- lambda- *ilvG*- *rfb*-50 *rph*-1) with chromosomal *tetR*, (spec^R^).	This study

U424	U423 +pZE11 +*P*_*araBAD *_reporter plasmid (spec^R^, amp^R^, kan^R^).	This study

U425	U423 with Δ*araC *chromosomal deletion (spec^R^).	This study

U426	U425 +pZE11-*araC *+*P*_*araBAD *_reporter plasmid (spec^R^, amp^R^, kan^R^).	This study

U427	U423 +pZE11 +pUA66 (spec^R^, amp^R^, kan^R^).	This study

U428	MG1655 (F- lambda- *ilvG*- *rfb*-50 *rph*-1) + *P*_*araC *_reporter plasmid (kan^R^).	[40]

U429	MG1655 (F- lambda- *ilvG*- *rfb*-50 *rph*-1) + *P*_*araBAD *_reporter plasmid (kan^R^).	[40]

U66	MG1655 (F- lambda- *ilvG*- *rfb*-50 *rph*-1) + pUA66 (kan^R^).	[40]

GFP reporter plasmids (pUA66 based [40], sc101 *ori*, kan^R^, with *gfpmut2 *[44]) for the *araC *(coordinates 69973-> 70469) and *araBAD *(coordinates 70469-> 69973) promoters are from the fluorescent reporter library given in detail in [40]. In short: the intergenic region between *araBAD *and *araC *(Figure 1d), with more than 100 bps of both flanking regions, was incorporated twice into the GFP reporter plasmid: once in the plus strand orientation (*araC *promoter) and once in the minus strand orientation (*araBAD *promoter).

*araC *was decoupled from its native regulation by cloning it into the pZE11 plasmid (colE1 *ori*, amp^R^, *P*_*LtetO-1 *_[45]) using the *Kpn*I and *Hind*III restriction enzymes. The *araC *gene (the entire coding region) was PCR amplified from MG1655 genomic DNA with the following start and end coordinates: 70387-71299 (positive strand), by using the following primers: 5' ggcggtaccatggctgaagcgcaaaatgatcc for the 5' end and 5' ggcaagcttccgtcaagccgtcaattgtctg for the 3' end. The PCR product and the pZE11 plasmid were digested with *Kpn*I and *Hind*III, and then were ligated, yielding pZE11-*araC*. A self-ligated pZE11 was generated as well to serve as a control plasmid.

### Strains (see Table 1)

In order to achieve maximal genotypic identity between the wild-type (with NAR) and the mutant (without NAR) strains, a modified wild-type strain was constructed. *tetR *gene (z1, spec^R^) from DH5αZ1 was P1 transduced into the wild-type MG1655 chromosome (K12 strain MG1655: F- lambda- *ilvG*- *rfb*-50 *rph*-1), yielding strain U423 (MG1655z1). The pZE11 plasmid and the *araBAD *reporter plasmid were transformed into U423, yielding strain U424.

An isogenic Δ*araC *strain was obtained by deleting *araC *from the MG1655z1 chromosome (coordinates 70391-> 71244) using the phage λ Red recombination system [46,47], yielding strain U425 (MGz1Δ*araC)*. Primers 5' ggacaattggtttcttctctgaatggtgggagtatgaaaagtatggtgtaggctggagctgcttc 3' (for the 5 prime end) and 5' gccgtcaattgtctgattcgttaccaattatgacaacttgacggctaccatatgaatatcctccttag 3' (for the 3 prime end) were used to amplify the kanamycin resistance gene from the pKD4 plasmid with extensions homologous to the 5' and 3' ends of the *araC *gene, to allow recombination. Kanamycin resistance was removed from the deleted strain using FLP recombinase, as described [47]. U425 did not grow on L-Arabinose as a sole carbon source. The *araC *deletion and the integrity of the *araC*\*araBAD *divergent chromosomal promoter were verified using PCR and sequencing of the scar region. *araBAD *reporter and pZE11-*araC *plasmids were transformed into U425, yielding strain U426. Transformation of pZE11-*araC *into the U425 strain restored its ability to grow on L-Arabinose as a sole carbon source. This strain produced AraC levels, similar to that of the wild-type strain (assessed from the promoter activity of the *araBAD *reporter at maximal induction, which was about 70% of that of the wild-type strain).

MG1655z1 with empty-pZE11 and pUA66 promoterless reporter plasmid (strain U427) was used for fluorescence background subtraction for U424 & U426.

Strain U66 [35,40] was used for fluorescence background subtraction for U428.

### Growth conditions and measurements

Strains were grown over-night in M9 minimal medium containing 0.4% glucose 0.05% casamino acids, 50 μg/ml kanamycin and 100 μg/ml ampicillin (dictated by the plasmids in each strain) at 37°C. No aTc was used to induce pZE11-*araC*, since its basal expression level was found to be close to the wild type AraC level. Using a robotic liquid handler (Freedom Evo, Tecan), flat bottom 96-well plates (Nunc) were prepared with 150 μl of M9 minimal medium containing 0.2% glucose 0.05% casamino acids, 30 mM cAMP, 50 μg/ml ampicillin and 25 μg/ml. L-Arabinose, in increasing concentrations was added. The wells were inoculated with the reporter strain at a 1:600 dilution from the overnight culture. Wells were then covered with 100 μl of mineral oil (Sigma) to prevent evaporation (a step which we previously found not to significantly affect aeration or growth [48,49], and transferred into an automated incubator. Cells were grown in an incubator with shaking (6 hz) at 30°C for about 20 hr. Every 8 minutes the plate was transferred by the robotic arm into a multi-well fluorimeter (Infinite F200, Tecan) that read OD (600 nm) and GFP fluorescence (535 nm).

### Data analysis

Promoter activity for each well was calculated from the OD and GFP measurements after subtracting the OD and GFP backgrounds. GFP background was obtained for each well from the promoterless control strains U427 (for strains U424 and U426) and U66 (for strain U428) (Additional File 1, Figure S1). Promoter activity was calculated by computing the rate of accumulation of GFP per unit time divided by the OD (dGFP/dt/OD) as described [49].

## Abbreviations

amp^R^: ampicillin resistance; cAMP: cyclic adenosine mono phosphate; CRP: cAMP receptor protein; aTc: anhydrotetracycline; GFP: green fluorescent protein; kan^R^: kanamycin resistance; NAR: negative auto-regulation; OD: optical density; PA: promoter activity; s.e.: standard error; spec^R^: spectinomycin resistance; TF: transcription factor.

## Competing interests

The authors declare that they have no competing interests.

## Authors' contributions

DM designed the research, preformed the molecular genetic manipulations and the experiments, analyzed data and wrote the paper. ED designed the research, created the mathematical model, analyzed data and wrote the paper. AB designed the research, preformed the molecular genetic manipulations, analyzed data and wrote the paper. UA designed the research, analyzed data and wrote the paper. All authors read and approved the final manuscript.

## Supplementary Material

Additional file 1 **Supplementary on-line material. Detailed model of the arabinose system and examples for raw data figures**. Click here for file
