# Supplementary material for: Negative auto-regulation increases the input dynamic-range of the arabinose system of Escherichia coli
Source: BMC Syst Biol. 2011 Jul 12;5:111. doi: 10.1186/1752-0509-5-111 (PMC3163201; doi:10.1186/1752-0509-5-111)
Supplement: Additional file 1 — Supplementary on-line material. Detailed model of the arabinose system and examples for raw data figures. [file 1752-0509-5-111-S1.DOC]

**Negative auto-regulation increases the input dynamic-range of the arabinose system of *Escherichia coli***

Daniel Madar, Erez Dekel, Anat Bren and Uri Alon

*Department of Molecular Cell Biology, The Weizmann Institute of Science, Rehovot, 76100, Israel.*

**Supplementary on-line material**

**Table of content:**

1. The raw *araBAD* promoter activity as measured by GFP fused to *P_araBAD_* promoter is high above background level, p. 2-3
2. Bacterial growth curve, p. 4
3. A detailed model for the regulation of the arabinose system by AraC, p. 5-7
4. The half-induction point of the input function shifts according to the downstream gene regulation, p. 8-9
5. References, p. 10

**The raw *araBAD* promoter activity as measured by *gfp* fused to *P_araBAD_* promoter is high above background level**

Our experimental procedure includes measurements of the fluorescence from a *gfp* gene fused to the native *araBAD* promoter. We use a bright, non-toxic, rapidly folding variant of GFP called *gfpmut2* [1]. This protein fluoresces at approximately the same wavelength as GFP. Here we show that the measured GFP levels are high above the background florescence levels as was measured from a control strain (U427) with the same *gfp* gene, but without a promoter (promoterless). Promoter activity signal to background ratios greater than 10 for L-Arabinose levels greater than 0.57 mM, for the *araBAD* reporter (Fig. S1c). This shows that the measured induction of GFP levels is due to the promoter activity of *araBAD*, and not an induction of background fluorescence.

**Figure S1**: **GFP levels of the *araBAD* promoter is high above the background.** (**a**) Raw GFP levels over different arabinose concentrations of the parental strain with *araBAD* promoter fused to a *gfp* gene (U424- green circles), compared to the same strain with a promoter-less *gfp* (U427- blue circles). Both curves are average over 3 repeats. The error bars indicate the s.d. (**b**) & (**c**) are Promoter activity (dGFP/dt/OD) of *araBAD* (strain U424- dashed blue line) over time compared to the promoterless strain (U427- solid pink line). The results are from typical wells. The dotted black vertical lines show the time window from which the results were taken (5-7 hours after inoculation of 1:600 dilution from overnight incubation). (**b**) is at high arabinose level (55 mM) and (**c**) is at low arabinose level (0.57 mM).

**Bacterial growth curve**

**Figure S**2 – **Bacterial growth curve.** Shown is the growth curve of *E. coli* in a typical condition of 4 mM L-Arabinose, in a 96-wells plate. The dotted black vertical lines show the time window from which the results were taken (5-7 hours after inoculation of 1:600 dilution from overnight incubation, in the middle of the exponential phase).

**Detailed model for the regulation of the arabinose system by AraC**

We developed a mass-action mathematical model based on the *araC* regulation system. A transcription factor (e.g AraC), whose concentration is denoted *x*, binds internal arabinose (s) with dissociation constant *K_s_*. The amount of *x* bound to *s*, which is the active form of the transcription factor, is given by:

(A1) $[xs]=\frac{x_{f}s}{K_{s}}$

Where *x_f_* is the free transcription factor. The transcription factor (*x*) can be negatively auto-regulated, and in this case, its level depends on the inducer (e.g. arabinose) concentration.

Assuming conservation of the transcription factor:

(A2) $[x_{T}]=x=[xs]+[x_{f}]$

Where *x_T_* or *x* denote the total transcription factor concentration. From equations (A1) and (A2) we get:

(A3) $[xs]=\frac{xs}{K_{s}+s}$

The binding of the transcription factor and inducer to the promoter is described by an equation similar to Eq. (A1):

(A4) $[xsp]=\frac{[p_{f}][xs]}{K_{a}}$
Where *p_f_* is the free promoter concentration and *K_a_* is the dissociation constant of the complex [*xs_i_*] to the promoter (e.g. *araBAD* promoter). The binding of transcription factor without inducer to the promoter is:

(A5) $[xp]=\frac{[p_{f}][x_{f}]}{K_{r}}$
Where *K_r_* is the dissociation constant of free transcription factor and the promoter.

For the case of a single promoter:

(A6) $[p_{T}]=1=[xs_{i}p]+[xp]+[p_{f}]$

The concentration of the bound promoter is obtained by substituting Eq. (A6) into (A4) and (A5):

(A7) $[xsp]=\frac{\frac{xs}{K_{s}K_{a}}}{1+\frac{s}{K_{s}}+\frac{x}{K_{r}}+\frac{xs}{K_{s}K_{a}}}$

Cooperativity is introduced by assuming that several (*n*) transcription factors cooperatively binds and regulate the promoter [2]. The promoter activity is the sum of the free and bound promoter’s activities:

(A8) $\beta_{z}=\frac{\beta_{z}^{(1)}\left(1+\frac{s}{K_{s}}\right)^{n}+\beta_{z}^{(2)}\left(\frac{xs}{K_{s}K_{a}}\right)^{n}}{\left(1+\frac{s}{K_{s}}\right)^{n}+\left(\frac{x}{K_{r}}\right)^{n}+\left(\frac{xs}{K_{s}K_{a}}\right)^{n}}$
Where *β_z_*^(1)^ is the production rate when the promoter is free, and *β_z_*^(2)^ is the production rate when the promoter is bound. In this model one obtains an ideal activator in the limit:$K_{r}\to\infty$, $\beta_{z}^{(1)}\to0$, and an ideal repressor in the limit:$K_{a}\to\infty$, $\beta_{z}^{(2)}\to0$. Setting the values of *K_a_*, *K_r_*, *β_z_*^(1)^, *β_z_*^(2)^ to intermediate levels results in a dual repressor-activator regulation.

If the transcription factor is also negatively auto-regulated we can write at steady state:

(A9) $x=x_{st}=\frac{\beta_{x}}{\alpha}\frac{1+\frac{s}{K_{s}}}{1+\frac{s}{K_{s}}+\frac{x}{K_{xr}}}$
Where *α* is the dilution/degradation of *x*.

The above equation can be solved analytically and the level of the transcription factor (*x*) can be substituted into the equation for the promoter activity (Eq. A8).

We fitted this set of equations to the experimental data. Although there are many solutions to the fitted input function (Eq. A8), all the solutions have common characteristics. We find that all of the computed input functions are similar to the measured input function of *araBAD* by acting as a repressor at low inducer levels and as an activator at high levels.

**The half-induction point of the input function shifts according to the downstream gene regulation**

Comparing the measured induction curves in Fig. 3 in the MS we note that the half-induction point of the input function without NAR, denoted *K_noNAR_*, (Fig. 3b in MS, red curve) is right shifted with respect to the half-induction point of the input function with NAR, denoted *K_NAR_* (Fig. 3a in MS, blue curve). We ask what determines the extent and direction of this shift. To investigate this we used our detailed model (p. 5-7 in the SOM) to calculate this shift. We found that if the TF level without NAR is above the TF level at half induction with NAR then the *K_noNAR_* shifts to higher values if the TF acts as a repressor and to lower values if it acts as an activator. The shifts are in opposite directions if the TF level without NAR is below the TF level at half induction with NAR. If the TF is a perfect dual regulator then there will be no shift of its downstream gene (Fig. S3). This can be explained intuitively: Let us assume that the TF is a repressor. The expression level of *Z*, at a given level of the inducer (*s*) will be higher with NAR since the TF concentration with NAR is lower than the TF concentration without NAR. This intuition also explains the increase dynamic range of NAR.

The shift of *K_noNAR_* with respect to *K_NAR_* in the arabinose system was to higher values, and the level of AraC without NAR was measured to be at the maximal level of AraC with NAR. From the directionality and extent of the shift and from the AraC levels, we can calculate that AraC regulates *araBAD* as a dual regulator which is 25% more repressor-like (cyan dot in Fig. S3).

**Figure S3**: **Shift of the half-induction point of input function as a function of regulation type**. The ratio between the half induction point without NAR to the half-induction point with NAR (*K_noNAR_ /K_NAR_*) as a function of the regulation type of the TF (*X*). Regulation type of -1 indicates a pure repressor and 1 indicates pure activator. Regulation type of 0 indicates a perfect dual regulation in which the repressor and activator are fully symmetric. The solid blue line shows the calculated shift of *K_noNAR_ /K_NAR_* as a function of regulation type. *K_noNAR_ /K_NAR_* will shift to lower values if the regulation is ‘activator-like’ and to higher values if the regulation is ‘repressor like’. The cyan point indicates the measured shift in the *ara* system. The shift indicates that AraC is not a perfect dual regulator, but is more a repressor than an activator.

**References**

1. Cormack BP, Valdivia RH, Falkow S: **FACS-optimized mutants of the green fluorescent protein (GFP)**. *Gene* 1996, **173**:33-38.

2. Bintu L, Buchler NE, Garcia HG, Gerland U, Hwa T, Kondev J, Kuhlman T, Phillips R: **Transcriptional regulation by the numbers: applications**. *Curr. Opin. Genet. Dev* 2005, **15**:125-135.
